# Comprehensive Analysis of Macrophage Dynamics, CCBE1, and Their Implications in Colorectal Cancer Microenvironment: Insights Into Tumor Progression and Therapeutic Opportunities

**DOI:** 10.1155/genr/2678696

**Published:** 2026-07-01

**Authors:** XiaoFei Fan, ChunYan Zhang, RongRong Gu, Xin Xu

**Affiliations:** ^1^ Department of Pharmacy, Affiliated Hospital of Nantong University, Nantong, 226001, Jiangsu, China, ahnmc.com

**Keywords:** CCBE1, colorectal cancer, immune, macrophage

## Abstract

**Background:**

The significance of M2 macrophages in cancer is well established, yet their specific role and the regulatory molecules involved in colorectal cancer remain unclear.

**Methods:**

Using publicly available single‐cell RNA‐seq data from four colorectal cancer datasets (EMTAB8107, GSE139555, GSE146771, and GSE166555), we evaluated macrophage proportions and their functional enrichment. Immune cell infiltration was quantified by CIBERSORT, quanTIseq, and xCell on TCGA‐COAD bulk transcriptomes, with immune correlations adjusted for tumor purity. GSEA was performed with MSigDB v7.4 gene sets (Hallmark, GO, KEGG). Prognostic models were built using Kaplan–Meier and multivariate Cox regression. In vitro, CCBE1 was silenced in SW480 and HCT116 cells; proliferation was measured by CCK‐8, colony formation, and EdU assays, and migration/invasion were measured by transwell assays. M2 polarization was assessed by flow cytometry.

**Results:**

Macrophages represented a substantial fraction (20%–35% across datasets) of the tumor microenvironment and were associated with upregulated coagulation and KRAS signaling. Consensus M2 macrophage infiltration was linked to 91 upregulated genes, among which CCBE1 emerged as an independent prognostic risk factor (multivariate Cox: HR = 1.305 [95% CI: 1.039–1.639], *p* < 0.05). CCBE1 was overexpressed in colorectal cancer cell lines compared to NCM460. Silencing CCBE1 significantly suppressed cell proliferation, migration, and invasion. Moreover, the conditioned medium from CCBE1‐knockdown cancer cells decreased the proportion of CD206+ M2 macrophages and upregulated M1 markers while downregulating M2 markers, indicating that CCBE1 promotes M2 polarization.

**Conclusions:**

CCBE1 is an oncogenic driver in colorectal cancer that independently predicts poor survival and functionally enhances tumor cell proliferation, invasion, and M2 macrophage polarization. Targeting CCBE1 may represent a potential therapeutic strategy for colorectal cancer.

## 1. Introduction

Colorectal cancer is a common malignancy with rising global incidence, yet current treatments remain limited for advanced disease [1, 2]. The tumor microenvironment plays a central role in the initiation, progression, and treatment resistance of colorectal cancer, in which the interaction between immune cells and tumor cells plays an important role [3, 4]. Among these, M2‐polarized macrophages play a particularly critical role in tumor progression: they promote tumor growth, invasion, and immune evasion by secreting growth factors, suppressing antitumor immunity, and promoting angiogenesis [5, 6]. Several studies have preliminarily revealed the promoting role of M2 macrophages in colorectal cancer [7–10]. However, the molecules that influence M2 macrophage infiltration in colorectal cancer remain largely unclear.

M2 macrophage polarization reflects a functionally distinct immunosuppressive program, rather than simply an increase in macrophage abundance. While previous studies have largely reported associations between M2 infiltration and prognosis, it remains unclear whether tumor‐derived molecules actively drive M2 polarization and whether they carry functional significance beyond such correlative patterns. In this study, we combined single‐cell and bulk transcriptomic analyses with consensus‐based immune deconvolution to quantify M2 infiltration and applied differential expression analysis, LASSO regression, and multivariable Cox regression to systematically identify candidates with both prognostic relevance and potential functional involvement in M2 biology.

Through this screening framework, we identified CCBE1 as an M2 macrophage‐associated molecule that independently correlates with patient survival. Our analyses revealed that macrophages constitute a substantial proportion of cells in the colorectal cancer microenvironment and are functionally coupled with coagulation, complement, and KRAS signaling pathways. CCBE1 exhibited a paradoxical expression pattern, downregulated in bulk tumor tissue yet upregulated in cancer cell lines, which we resolved by demonstrating that bulk tissue CCBE1 signal predominantly originates from stromal and endothelial compartments and is diluted by tumor cells. Functionally, CCBE1 knockdown suppressed colorectal cancer cell proliferation, invasion, and migration in vitro, and the conditioned medium (CM) from CCBE1‐knockdown cells reduced M2 polarization while upregulating M1 markers. These results establish CCBE1 as a molecule that simultaneously enhances tumor cell aggressiveness and promotes an M2‐skewed immunosuppressive microenvironment, providing a candidate link between tumor‐derived signals and macrophage‐driven immune evasion in colorectal cancer.

## 2. Methods

### 2.1. Data Collection

Gene expression data (RNA‐seq) and corresponding clinical annotations for colorectal cancer were retrieved from The Cancer Genome Atlas (TCGA) colon adenocarcinoma (COAD) project via the UCSC Xena platform (https://xenabrowser.net/datapages/) [11, 12]. Only primary tumor samples with available survival information were retained; metastatic and recurrent specimens were excluded. No adjacent normal tissue samples from TCGA were included in the main immune deconvolution or differential expression analyses, as the focus of this study was on the tumor microenvironment. The final analytical cohort comprised 521 primary COAD samples. The original expression profiles, provided as “STAR‐Counts” from the UCSC Toil RNA‐seq Recompute compendium, were downloaded and preprocessed using custom R scripts (R Version 4.2.3). Counts were converted to transcripts per million (TPM) and subsequently log2(TPM+1) transformed. Gene‐level annotation was performed using the human reference genome file (GRCh38) from Ensembl (Version 108). Before analysis, genes with zero counts across all samples were removed, and potential batch effects were evaluated and found to be negligible across sequencing plates. No additional purity‐based sample filtering was applied; instead, tumor purity was accounted for in downstream correlation analyses using ESTIMATE‐derived scores. Clinical data (BCR‐XML format) were parsed to extract overall survival (OS) time, vital status, age, gender, and pathological stage, with records from patients with incomplete survival data excluded from prognostic modeling. Open‐access immunohistochemistry (IHC) and subcellular localization images were obtained from the Human Protein Atlas (HPA) database [13].

### 2.2. Differential Expression Analysis

Differentially expressed genes (DEGs) between the high and low M2 macrophage infiltration groups were identified using the limma package (Version 3.54.2) [14]. The input consisted of log2(TPM+1)‐normalized expression matrices. A linear model was fitted for each gene, and empirical Bayes moderation was applied. Genes with a |log2 fold change (log2FC)| > 1 and an adjusted *p* value (Benjamini–Hochberg method) < 0.05 were considered significantly differentially expressed.

### 2.3. Single‐Cell Analysis

In our single‐cell analysis segment, we utilize the TISCH (Tumor Immune Single‐Cell Hub) project for an extensive exploration of cellular heterogeneity at the single‐cell level through online analysis [15]. TISCH serves as a valuable resource for examining single‐cell RNA sequencing (scRNA‐seq) data across diverse cancer types. Leveraging TISCH, we employ computational methods to delineate distinct cell populations, identify cell types, and unveil transcriptional trajectories (https://tisch.compbio.cn/documentation/). This approach allows us to dissect the intricate interplay between tumor and immune cells, offering insights into the dynamics of the immune response within the tumor.

### 2.4. Quantification of M2 Macrophages and Immune Microenvironment

For the quantification of M2 macrophages and the immune microenvironment section of our study, we employ a comprehensive set of algorithms based on expression profiles to quantify immune cell populations, with a particular focus on M2 macrophages. The algorithms utilized include CIBERSORT, EPIC, MCPcounter, QUANTISEQ, TIMER, and xCell [16–21]. The immune function terms were quantified using the Gene Set Variation Analysis (GSVA) algorithm [22]. All deconvolution analyses were performed on log2(TPM+1)‐normalized gene expression matrices. CIBERSORT was executed with the LM22 signature and 1000 permutations; EPIC, MCPcounter, quanTIseq, TIMER, and xCell were run using their default built‐in signatures and parameters. To address the potential confounding effects of tumor purity and stromal content on immune infiltration correlations, we performed additional correlation analyses using the “Purity Adjustment” option provided by the TIMER3 project (https://compbio.cn/timer3/) on the TCGA‐COAD dataset [23], thereby evaluating the association between CCBE1 abundance and immune cell fractions after adjusting for tumor purity.

### 2.5. Biological Enrichment Analysis

We employed Gene Set Enrichment Analysis (GSEA) and ClueGO analysis to characterize the biological functions associated with our gene expression data [24, 25]. Specifically, the Hallmark v7.5.1, C5 GO (all) v7.5.1, and C2 KEGG v7.5.1 gene sets were used for the analysis. Genes were preranked using the signal‐to‐noise ratio metric, and enrichment significance was determined through 1000 gene set permutations. Gene sets were considered significantly enriched at a false discovery rate (FDR) *q* value < 0.25. ClueGO analysis (v2.5.9, Cytoscape plug‐in) was conducted to identify overrepresented functional terms in biological ontologies and pathways within a given gene list. Enrichment was determined using a two‐sided hypergeometric test, and the Benjamini–Hochberg procedure was applied to correct for multiple testing. Terms with an adjusted *p* value (p.adj) < 0.05 were considered statistically significant.

### 2.6. Prognosis Analysis

We conducted a systematic investigation into the predictive factors influencing patient outcomes. OS was used as the primary endpoint, defined as the time from initial diagnosis to death from any cause. Patients alive at the last follow‐up or lost to follow‐up were censored at that time point. For Kaplan–Meier (KM) survival analysis, patients were stratified into “high” and “low” groups based on the median CCBE1 expression, where the optimal cutpoint was determined using the surv_cutpoint function in the survminer R package (Version 0.4.9), which identifies the threshold that best separates survival outcomes [26]. Univariate Cox regression analysis was utilized to assess the impact of individual prognostic factors on survival, providing hazard ratios and 95% confidence intervals (CIs) and identifying key determinants of patient outcomes. Multivariable Cox proportional hazards regression models were constructed, adjusting for age at diagnosis, sex, and pathological stage (AJCC Stage I–IV) when available, to determine whether CCBE1 remained an independent prognostic factor. Furthermore, LASSO regression analysis was employed using the glmnet package (version 4.1‐7) as an additional dimensionality‐reduction step to narrow down the most informative and robust candidate genes from the M2 macrophage‐associated molecules, thereby focusing subsequent analyses on the most research‐worthy target. All survival analyses were implemented in R (Version 4.2.3) with the survival (Version 3.5‐5), survminer (Version 0.4.9), glmnet (Version 4.1‐7), and rms (Version 6.7‐1) packages.

### 2.7. Cell Lines

We employed a diverse panel of cell lines, including NCM460, SW480, HCT116, and DLD‐1, which were purchased from the Cell Bank of the Shanghai Academy of Chinese Sciences. The human monocyte cell line THP‐1 was obtained from the American Type Culture Collection (ATCC, Manassas, VA). All cell lines were cultured under standard conditions: maintained in a humidified incubator at 37°C with 5% CO_2_. All culture media were supplemented with 10% fetal bovine serum (FBS) and 1% penicillin/streptomycin, with the exception that THP‐1 medium additionally contained 0.05 mM β‐mercaptoethanol. The basal media used for each line were as follows: DMEM for NCM460; RPMI‐1640 for SW480; McCoy’s 5A for HCT116 and DLD‐1; and RPMI‐1640 for THP‐1. Regular monitoring of cell morphology, viability, and passage number was conducted to ensure optimal conditions for experimental consistency.

### 2.8. Real‐Time Quantitative PCR (qPCR)

RNA extraction was performed using TRIzol reagent (Invitrogen, Carlsbad, CA, USA), and cDNA synthesis was carried out using the PrimeScript RT Reagent Kit (Takara, Tokyo, Japan). The subsequent analysis involved qRT‐PCR using the SYBR Green system, with GAPDH serving as the internal standard. The quantification of relative RNA expression levels was achieved using the 2^–ΔΔCT^ method. The primers used were as follows: CCBE1, forward, 5′‐AAGTCTTCAGGCGAGCTCACC‐3′; reverse, 5′‐GTTGTCCGTGCACTGCTGTTC‐3′; GAPDH, forward, 5′‐GCACCGTCAAGGCTGAGAAC‐3′, reverse, 5′‐TGGTGAAGACGCCAGTGGA‐3′.

### 2.9. Cell Transfection and Lentivirus Packaging

Lentiviral particles were produced using a third‐generation packaging system. For each transfection, 293T cells were cotransfected with three plasmids: the lentiviral packaging plasmid psPAX2, the envelope plasmid pMD2.G, and the transfer plasmid pLKO.1‐puro carrying the shRNA sequence, using Lipofectamine 2000 (Invitrogen) according to the manufacturer’s protocol. Two independent shRNA sequences targeting CCBE1 were used: sh‐CCBE1#1 (target sequence: CCATGAGAAGTCTGAGAACAT) and sh‐CCBE1#2 (target sequence: GAAGCCATACTGTCTGGATAT), along with a nontargeting control shRNA. Following transfection, the 293T cells were allowed to produce lentiviral particles. At 48 h post‐transfection, the supernatant containing lentiviral particles was collected and filtered through a 0.45‐μm membrane. Target cells were transduced with the lentiviral supernatant in the presence of 8 μg/mL polybrene. At 48 h postinfection, stably transduced cells were selected with 2 μg/mL puromycin, and knockdown efficiency was verified by qPCR.

### 2.10. Cell Proliferation Assay

We employed a multifaceted approach to assess cellular growth and replication. Cell Counting Kit‐8 (CCK8) assays were conducted to measure cell viability and proliferation based on the metabolic activity of cells. Briefly, cells were seeded at 2 × 10^3^ cells/well in 96‐well plates, and CCK‐8 reagent (Dojindo, Kumamoto, Japan) was added at 0, 24, 48, 72, and 96 h. Absorbance was measured at 450 nm using a microplate reader. For clone formation assays, cells were seeded at 500 cells/well in six‐well plates and allowed to form colonies for 14 days. Colonies were fixed with 4% paraformaldehyde, stained with 0.1% crystal violet, and manually counted. In addition, 5‐ethynyl‐2′‐deoxyuridine (EdU) incorporation assays were conducted to specifically label actively proliferating cells during DNA synthesis. Cells were incubated with 10 μM EdU for 2 h, and detection was performed using the Click‐iT EdU Imaging Kit (Invitrogen). The percentage of EdU‐positive cells was quantified from five random fields per well.

### 2.11. Transwell Assay

We employed a transwell assay to assess cell migration and invasion capabilities. Transwell inserts with 8‐μm pore size permeable membranes were utilized to create an upper and lower chamber. For migration assays, cells were serum‐starved for 12 h, and 5 × 10^4^ cells in a 200‐μL serum‐free medium were seeded into the upper chamber, and the lower chamber contained a medium with 10% FBS as a chemoattractant. After 24‐h incubation, nonmigratory cells on the upper surface were removed with a cotton swab. For invasion assays, the upper chamber was coated with Matrigel (Corning, 1:8 dilution), creating a barrier that cells needed to invade. After 48‐h incubation, migrated or invaded cells on the lower surface were fixed with 4% paraformaldehyde, stained with 0.1% crystal violet, and counted from five random fields per insert.

### 2.12. Immunofluorescence

For the immunofluorescence, cells were fixed with paraformaldehyde, followed by permeabilization with Triton X‐100 to enable antibody penetration. Blocking nonspecific binding sites was achieved using a BSA‐containing solution. Subsequently, cells underwent incubation with primary antibodies: anti‐CD68 (Abcam, ab955, 1:200) and anti‐CD206 (Abcam, ab64693, 1:200), were washed to remove unbound antibodies, and were then exposed to fluorochrome‐conjugated secondary antibodies. Nuclear staining was performed with DAPI, and cells were mounted using an appropriate medium. Visualization of immunofluorescent signals was conducted using a fluorescence microscope.

### 2.13. Macrophage Induction From Monocytes and Flow Cytometry

THP‐1 human monocytes (undifferentiated baseline state) were cultured in six‐well plates at a density of 5 × 10^5^ cells/well in an RPMI‐1640 medium containing 10% FBS, 1% penicillin/streptomycin, and 0.05 mM β‐mercaptoethanol. Macrophage differentiation was induced by treating cells with 100 ng/mL phorbol‐12‐myristate‐13‐acetate (PMA; Sigma‐Aldrich, Catalog No. P1585) for 48 h. After differentiation, cells were washed twice with PBS to remove PMA and cultured in a fresh PMA‐free complete medium for an additional 3 days to obtain resting M0 macrophages. For M2 polarization experiments, M0 macrophages were incubated with CM collected from control or CCBE1‐knockdown colorectal cancer cells (SW480 or HCT116) for 48 h. CM was prepared by culturing cancer cells in serum‐free RPMI‐1640 for 24 h, followed by centrifugation (300 × g, 5 min) and filtration (0.22 μm) to remove cell debris. To identify macrophage surface markers, cells were harvested, washed with chilled PBS, and incubated with anti‐CD206 (clone 19.2, FITC‐conjugated, BioLegend) or anti‐HLA‐DR (clone L243, PE‐conjugated, eBioscience) antibodies for 30 min at 4°C. After incubation, cells were washed twice with cold PBS and resuspended in flow cytometry staining buffer. The expression of CD206 and HLA‐DR on the macrophage surface was analyzed using a BD Accuri C6 flow cytometer (BD Biosciences, San Jose, CA, USA). The gating strategy was as follows: debris and doublets were excluded based on forward scatter (FSC) and side scatter (SSC) parameters; single live cells were then gated. Within this population, macrophages positive for CD206 or HLA‐DR were identified using gates set based on the corresponding isotype controls, with positivity defined as fluorescence intensity exceeding that of 99% of cells stained with the isotype control antibody. Data analysis was performed using BD Accuri C6 software and R (Version 4.2.3).

### 2.14. Statistical Analysis

All in vitro experiments were performed with at least three independent biological replicates (*n* = 3), and data are expressed as mean ± standard deviation (SD). For CCK8 assays, differences between groups over time were assessed by two‐way ANOVA with Bonferroni correction for multiple comparisons. For colony formation, EdU, and transwell assays, pairwise comparisons between two groups were performed using Student’s *t* test. The normality of the data distribution was assessed using the Shapiro–Wilk test. Parametric tests, such as Student’s *t* test for two‐group comparisons or one‐way ANOVA for multiple groups, were applied to normally distributed data. Nonparametric tests, including the Mann–Whitney *U* test and Kruskal–Wallis test, were used for non‐normally distributed data. Categorical data were analyzed using the chi‐square test or Fisher’s exact test. Correlation analysis, employing Pearson’s or Spearman’s rank correlation methods, was conducted based on the nature of the data distribution. To address multiple comparisons, the Bonferroni correction or FDR methods were applied. Survival analysis involved Kaplan–Meier curves and log‐rank tests, while Cox proportional hazards regression models were used for multivariate survival analysis. The significance threshold was set at a *p* value less than 0.05 for all tests. The entire statistical analysis, including data manipulation, was performed using the R programming language and SPSS software.

## 3. Results

### 3.1. Role of Macrophages in Colorectal Cancer Microenvironment

**FIGURE 1:**
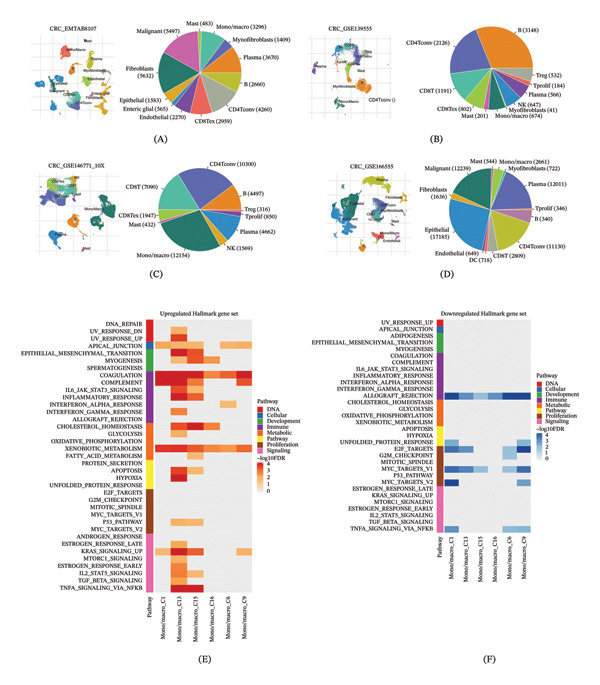
Macrophage/monocyte distribution in colon cancer datasets. (A–D) Graphical representation of macrophage/monocyte ratios in four single‐cell datasets (EMTAB8107, GSE139555, GSE146771, and GSE166555) related to colon cancer. (E) Hallmark enrichment analysis showing upregulated pathways associated with macrophage/monocyte. (F) Hallmark enrichment analysis depicting downregulated pathways associated with macrophage/monocyte.

Macrophages play a crucial regulatory role in the tumor microenvironment of colorectal cancer [27]. Initially, we examined macrophage/monocyte ratios in four single‐cell datasets related to colorectal cancer (EMTAB8107, GSE139555, GSE146771, and GSE166555) as depicted in Figure 1A–D [28–30]. Our findings revealed a substantial presence of macrophages in the colorectal cancer microenvironment, underscoring their significant biological relevance in this context. Moving forward, we delved into the biological functions of macrophages within the colorectal cancer microenvironment. Hallmark enrichment analysis demonstrated a correlation between the macrophage/monocyte presence and the upregulated activity of coagulation, complement, xenobiotic metabolism, KRAS signaling, and apical junction (Figure 1E). Conversely, there was a downregulation in the activity of allograft rejection, E2F targets, and MYC targets (Figure 1F).

### 3.2. Identification the Molecules Associated With M2 Macrophages and Their Biological Role

**FIGURE 2:**
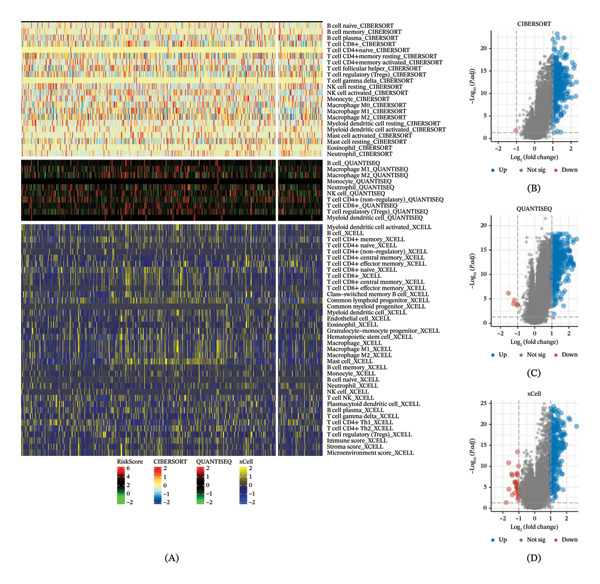
Identification of molecules associated with M2 macrophages infiltration in colon cancer. (A) CIBERSORT, QUANTISEQ, and XCELL algorithms were used for quantifying immune cell infiltrates in colon cancer. (B–D) DEG analysis graphs for patients with varying levels of M2 macrophage infiltration.

**FIGURE 3:**
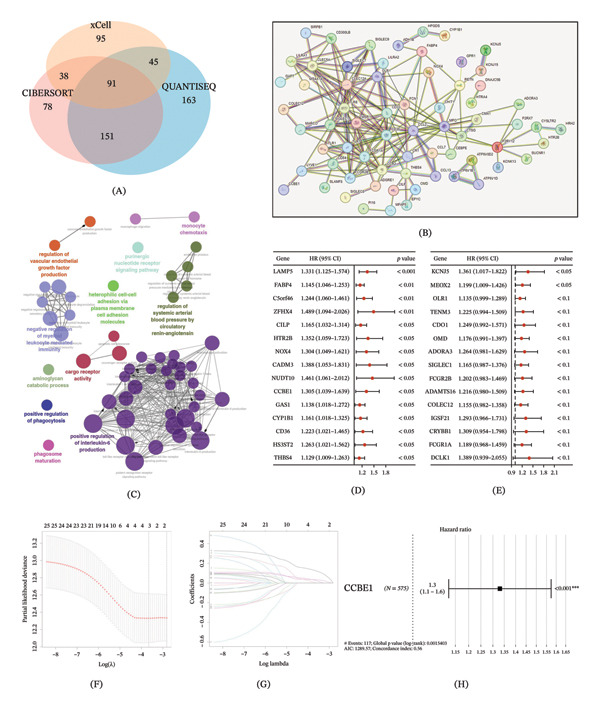
Biological impact and prognosis analysis of molecules associated with M2 macrophages. (A) Bar graph displaying 91 upregulated molecules identified in patients with high M2 macrophage infiltration. (B) Protein interaction network of molecules associated with M2 macrophages. (C) ClueGO analysis results showing pathways enriched in M2 macrophage‐related molecules. (D and E) Univariate Cox regression analysis identifying molecules significantly correlated with patient survival. (F and G) LASSO regression analysis used to reduce data dimensions. (H) Multivariable cox regression analysis identifying CCBE1 as a risk factor in colon cancer.

To uncover molecules associated with M2 macrophage infiltration, we employed CIBERSORT, QUANTISEQ, and xCell algorithms to quantify specific immune cell infiltrates from the transcriptional profiling data of colorectal cancer patients (Figure 2A). Among the results obtained, we identified M2 macrophages quantifiable by CIBERSORT, QUANTISEQ, and xCell algorithms (Figure S1). Subsequently, DEG analysis was conducted in patients with high and low M2 macrophage infiltration quantified by CIBERSORT, QUANTISEQ, and xCell algorithms, respectively (Figure 2B–D). Ultimately, 91 upregulated molecules were identified in patients with high M2 macrophage infiltration across the three algorithms (Figure 3A). The protein interaction network for these molecules is depicted in Figure 3B. ClueGO analysis revealed that these M2 macrophage‐related molecules were mainly associated with the regulation of vascular endothelial growth factor production, monocyte chemotaxis, purinergic nucleotide receptor signaling pathway, heterophilic cell–cell adhesion via plasma membrane cell adhesion molecules, regulation of systemic arterial blood pressure by circulatory renin‐angiotensin, cargo receptor activity, aminoglycan catabolic process, positive regulation of phagocytosis, phagosome maturation, and positive regulation of interleukin‐6 production (Figure 3C). Further analyses included univariate Cox regression to identify molecules significantly correlated with patient survival (Figure 3D,E), LASSO regression to reduce data dimensions (Figure 3F,G), and multivariable Cox regression to determine the final variable. The results highlighted CCBE1 as a risk factor for colorectal cancer patients (Figure 3H).

### 3.3. Expression Pattern of CCBE1 in Colorectal Cancer

**FIGURE 4:**
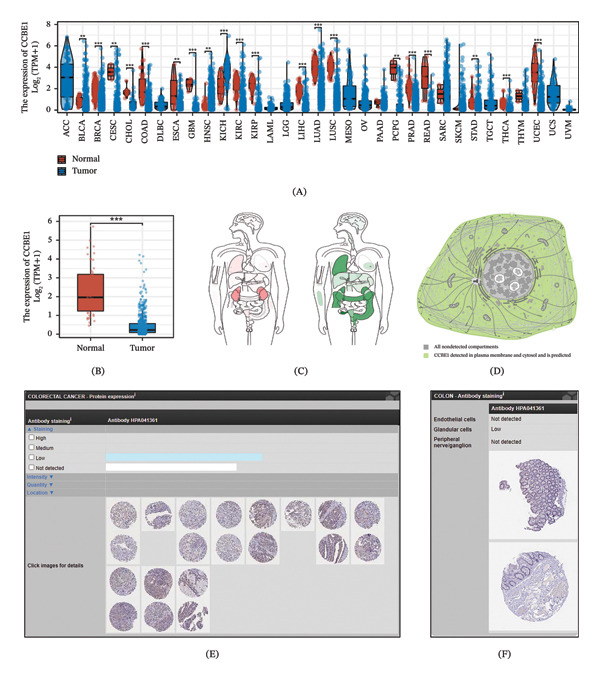
Expression and localization of CCBE1 across different cancer types. (A) Expression profile of CCBE1 across various cancer types, indicating differential expression. (B and C) Expression level of CCBE1 in colon cancer versus normal tissue (GEPIA database). (D) Subcellular localization data from the HPA database showing CCBE1 localization in the plasma membrane and cytosol. (E and F) Protein expression levels of CCBE1 in colorectal cancer and normal intestinal tissue.

Subsequently, we examined the expression profile of CCBE1 across various cancer types. The results revealed differential expression in many cancers, underscoring its potential involvement in tumorigenesis and development (Figure 4A). Notably, CCBE1 exhibited downregulation in colorectal cancer tissue (Figure 4B,C). Analyzing subcellular localization data from the HPA database, we observed that CCBE1 predominantly localizes to the plasma membrane and cytosol (Figure 4D). Furthermore, we noted that while the protein expression of CCBE1 is relatively low in both colorectal cancer and normal intestinal tissue, the protein expression level in colorectal cancer is higher than that in normal intestinal tissue (Figure 4E,F).

### 3.4. Biological Enrichment of CCBE1

**FIGURE 5:**
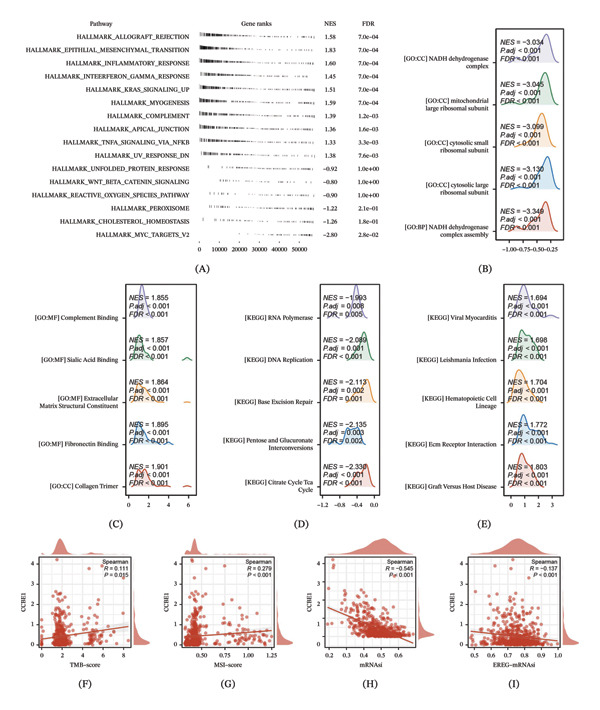
Biological enrichment analysis of CCBE1 in colon cancer. (A–C) GSEA highlighting the biological roles of CCBE1 in colon cancer, with a focus on hallmark and GO gene sets. (D and E) GSEA based on the KEGG gene set showing pathways influenced by CCBE1. (F–I) Correlation analysis of CCBE1 with TMB score, MSI score, and mRNA indices.

Utilizing the Hallmark gene set, we conducted GSEA analysis to elucidate the biological role of CCBE1 in colorectal cancer. The findings indicated that CCBE1 was associated with heightened activity in allograft rejection, epithelial–mesenchymal transition (EMT), inflammatory response, interferon‐gamma response, KRAS signaling, and myogenesis. Conversely, there was a lower activity observed in UV response, unfolded protein response, WNT beta‐catenin signaling, reactive oxygen species pathway, and peroxisome (Figure 5A). Expanding the analysis to the GO gene set, GSEA revealed that CCBE1 was correlated with decreased activity in NADH dehydrogenase complex, mitochondrial large ribosomal subunit, cytosolic small ribosomal subunit, cytosolic large ribosomal subunit, and NADH dehydrogenase complex assembly. Conversely, there was an increased activity observed in complement binding, sialic acid binding, extracellular matrix structural constituent, fibronectin binding, and collagen trimer (Figure 5B,C). In addition, GSEA analysis based on the KEGG gene set indicated that CCBE1 was correlated with reduced activity in RNA polymerase, DNA replication, base excision repair, pentose and glucuronate interconversions, and citrate cycle TCA cycle. On the other hand, there was heightened activity in viral myocarditis, leishmania infection, hematopoietic cell lineage, ECM receptor interaction, and graft‐versus‐host disease (Figure 5D,E). Moreover, we observed a positive correlation between CCBE1 and TMB score, MSI score, while a negative correlation with mRNAsi and EREG‐mRNAsi (Figure 5F–I).

### 3.5. Role of CCBE1 in Colorectal Cancer Microenvironment

**FIGURE 6:**
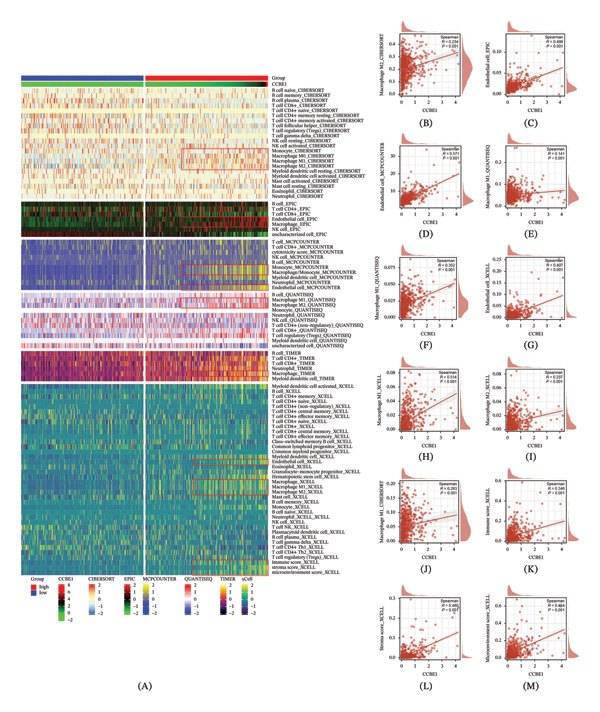
Correlation between CCBE1 expression and components of the colon cancer microenvironment. (A) Overview of various algorithms used to assess the colon cancer microenvironment. (B–M) Correlation analysis between CCBE1 expression and various microenvironment components.

Next, we investigated the impact of CCBE1 in the colorectal cancer microenvironment. Initially, we assessed the microenvironment of colorectal cancer using multiple algorithms, including CIBERSORT, EPIC, MCPcounter, QUANTISEQ, TIMER, and xCell (Figure 6A). From the quantified results, we observed a positive correlation between CCBE1 and various components of the microenvironment, including M2 macrophage_CIBERSORT, endothelial cell_EPIC, endothelial_MCPCOUNTER, M1 macrophage_QUANTISEQ, M2 macrophage_QUANTISEQ, endothelial_XCELL, M1 macrophage_XCELL, M2 macrophage_XCELL, and M1 macrophage_CIBERSORT (Figure 6B–J). After adjusting for tumor purity, CCBE1 expression remained significantly positively correlated with M2 macrophage infiltration (Figure S2). Meanwhile, we found that CCBE1 was significantly positively correlated with stromal and immune scores and significantly negatively correlated with tumor purity (Figure 6K–M and Figure S3).

### 3.6. CCBE1 Promotes Colorectal Cancer Proliferation and Metastasis

**FIGURE 7:**
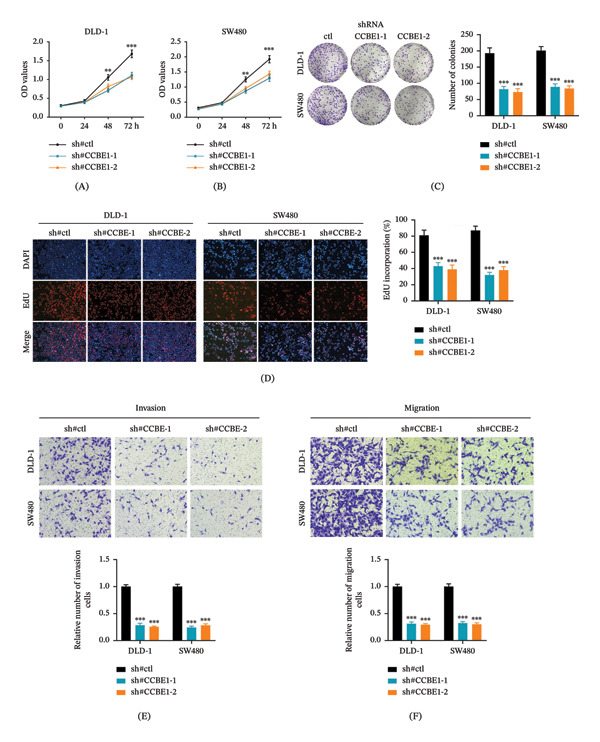
Impact of CCBE1 on proliferation and metastasis of colon cancer cells. (A–C) Proliferation assessments using CCK8 and colony formation assays. (D) EdU assay results indicating proliferation rates in colon cancer cells post‐CCBE1 knockdown. (E, F) Transwell assay results showing the effects of CCBE1 knockdown on invasion and migration abilities of colon cancer cells.

Subsequently, we examined the impact of CCBE1 on colorectal cancer cells. qPCR results revealed an upregulation of CCBE1 in colorectal cancer cells compared to normal NCM460 cells (Figure S4A). To explore the functional consequences of CCBE1, we performed knockdown experiments using two independent shRNAs targeting CCBE1 (sh‐CCBE1#1 and sh‐CCBE1#2), both of which effectively reduced CCBE1 expression (Figure S4B). CCK8 assay results demonstrated that CCBE1 knockdown significantly inhibited the proliferation of colorectal cancer cells, a finding corroborated by colony formation assay results (Figure 7A–C). The EdU assay revealed a lower EdU‐positive rate in colorectal cancers with CCBE1 knockdown compared to the control group (Figure 7D). Transwell assay results indicated that CCBE1 inhibition remarkably suppressed the invasion and migration abilities of colorectal cancer cells (Figure 7E,F).

### 3.7. CCBE1 Is Associated With the M2 Macrophage Polarization

**FIGURE 8:**
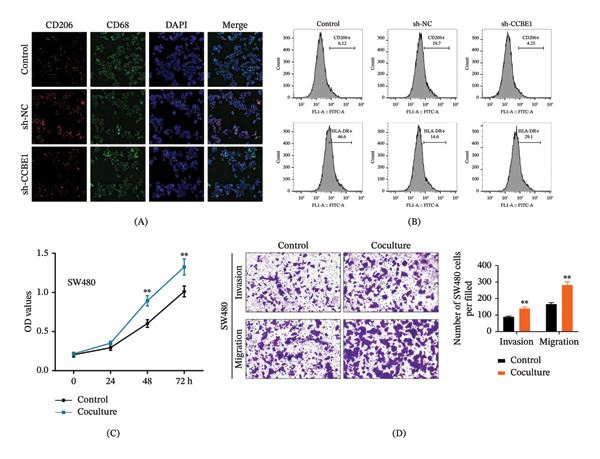
Effect of CCBE1 modulation on macrophage polarization and colon cancer cell dynamics. (A) Immunofluorescence assays demonstrating the ratio of CD206 positive cells in macrophages cocultured with supernatants from CCBE1 knockdown and control cells. (B) Expression analysis of M1 and M2 macrophage markers in macrophages cocultured with different levels of CCBE1. (C and D) Functional assays showing the impact of M2 macrophages on the proliferation, invasion, and migration abilities of colon cancer cells.

Building on the immune infiltration findings, we identified a correlation between CCBE1 and M2 macrophage infiltration. To validate the impact of CCBE1 in cancer cells on macrophages, we introduced the culture supernatants of CCBE1 knockdown and control intestinal cancer cells to M0 macrophages induced by THP‐1. Subsequently, immunofluorescence assays demonstrated that macrophages cocultured with supernatants from CCBE1 knockdown cells exhibited a lower ratio of CD206 positive cells (Figure 8A). As anticipated, M0 macrophages cocultured with lower CCBE1 levels displayed increased expression of M1 markers and decreased expression of M2 markers (Figure 8B). Continuing our investigation, we explored the impact of M2 macrophages on colorectal cancer cells. The results indicated that colorectal cancer cells cocultured with M2 macrophages tended to exhibit higher proliferation, invasion, and migration abilities (Figure 8C,D).

## 4. Discussion

Colorectal cancer is a prevalent malignant tumor arising from abnormal cell growth in the colorectal tissue [31]. The global incidence of colorectal cancer has been steadily increasing, posing a significant challenge in the field of public health [32, 33]. Despite some progress in colorectal cancer treatment, including surgical interventions, radiation therapy, and chemotherapy, notable limitations persist [34]. The tumor microenvironment plays a pivotal role in tumor initiation, progression, and response to treatment [35]. In addition, significant changes in cytokines are observed in colorectal cancer patients before and after surgery, supporting a dynamic interaction between tumor burden and the immune microenvironment [36]. Therefore, exploring the tumor microenvironment may provide new insights into individualized therapies and overcome prevailing treatment limitations.

In this study, we integrated single‐cell and bulk transcriptomic analyses to dissect the functional landscape of macrophages in colorectal cancer and to identify CCBE1 as an M2 macrophage‐associated molecule with prognostic significance. We found that macrophage presence is coupled with upregulated coagulation, complement, KRAS signaling, and apical junction pathways, a functional signature indicating that macrophages in the colorectal cancer microenvironment are not merely structural components but are actively engaged in shaping a procoagulant, proinflammatory, and stromal‐remodeling niche. Conversely, the suppression of allograft rejection and MYC/E2F target activities implies a concomitant dampening of certain adaptive immune and proliferative programs, potentially reflecting the immunosuppressive reconfiguration of the tumor ecosystem.

A notable finding in our study is the apparent discrepancy between tissue‐level and cell‐line‐level expression patterns of CCBE1. Specifically, while CCBE1 mRNA was downregulated in bulk colorectal cancer tissues compared with normal tissues in the TCGA cohort, it was upregulated in colorectal cancer cell lines, and its knockdown consistently suppressed malignant phenotypes. Our additional analyses suggest that this paradox can be resolved by a dual, compartment‐specific model. In bulk tumor tissue, the observed downregulation largely reflects the dilution of CCBE1‐expressing stromal cells within the tumor mass, which masks the higher cell‐intrinsic expression of CCBE1 in the cancer cell compartment. This interpretation is further supported by the positive correlation of CCBE1 with stromal infiltration scores and its negative correlation with tumor purity. Conversely, in isolated cancer cell lines, the stromal dilution effect is eliminated, unmasking the cell‐autonomous oncogenic functions of CCBE1. Thus, the net contribution of CCBE1 to tumor progression likely depends on the balance between its stromal reservoir and its upregulation in malignant epithelial cells, a balance that bulk transcriptomic data alone cannot fully deconvolve.

CCBE1, a collagen and calcium‐binding EGF domain‐containing protein encoded by the CCBE1 gene, participates in embryonic development and angiogenesis [37, 38]. In cancer, CCBE1 exhibits context‐dependent dual roles. In hepatocellular carcinoma, CCBE1 suppresses tumor progression by impeding the TGFβ‐DRP1 axis and promoting mitochondrial fusion [39], whereas in gastrointestinal stromal tumors, it facilitates angiogenesis and contributes to imatinib resistance [40]. In glioma, Hsa_circ_0076931 suppresses malignancy through the miR‐6760‐3p/CCBE1 axis [41]. Our study extends this functional network to colorectal cancer, where CCBE1 appears to exert a predominantly oncogenic influence.

Our biological enrichment analyses revealed that CCBE1 expression correlates with heightened activity of EMT, inflammatory response, interferon‐gamma response, and KRAS signaling pathways. The co‐occurrence of EMT signatures and the positive correlation between CCBE1 and M2 macrophage infiltration suggest that CCBE1 may coordinate tumor cell‐intrinsic invasive programs with paracrine signals that shape an M2‐skewed, immunosuppressive microenvironment. This interpretation is reinforced by our in vitro finding that CM from CCBE1‐knockdown cells reduced CD206^+^ M2 polarization while upregulating M1 markers, providing functional evidence that tumor cell‐derived CCBE1 acts, either directly or indirectly, on macrophage polarization. The existing literature supports the relevance of this EMT–immune axis in colorectal cancer: PCSK9 has been shown to promote EMT and macrophage polarization through PI3K/AKT signaling [7], while alternative splicing downstream of EMT amplifies phenotypic plasticity [42], and SPOCK1 drives proliferation and migration via NF‐κB‐mediated EMT induction [43]. CCBE1 may function within a similar signaling framework, possibly through integrin‐mediated ECM interactions or TGF‐β‐related pathways, although the precise molecular mechanisms remain to be elucidated.

Regarding the association between CCBE1 and endothelial–macrophage infiltration, our correlation analyses indicate that CCBE1 expression tracks with both endothelial and M2 macrophage abundance in the colorectal cancer microenvironment. This observation suggests that CCBE1 may be embedded within the endothelial–macrophage crosstalk axis that facilitates angiogenesis and immunosuppression. In this context, CCBE1 expressed in the stromal/endothelial compartment may contribute to a perivascular niche that promotes M2 polarization, while tumor cell‐intrinsic CCBE1 simultaneously drives invasive behavior. This dual compartment model, in which stromal CCBE1 supports a proangiogenic, M2‐permissive microenvironment and tumor cell CCBE1 enhances malignancy, provides a parsimonious explanation for the apparent tissue‐level downregulation versus cell line upregulation paradox. Future studies employing coculture systems, endothelial tube formation assays, and in vivo models will be necessary to experimentally dissect these compartment‐specific contributions.

Several limitations should be considered when interpreting our findings, as they may affect the robustness of our inferences. Our analyses rely on bulk transcriptomic data and computational deconvolution from public CRC cohorts, which introduces uncertainties related to tissue purity and unmeasured confounding. Tumor cell fraction and stromal heterogeneity may bias the inferred CCBE1–macrophage associations. Algorithmic uncertainty is also inherent to deconvolution; different strategies yield variable estimates of immune cell fractions, and although we compared multiple algorithms and interpreted cross‐algorithm consistency cautiously, residual batch effects and reference‐matrix limitations remain. Most critically, the lack of in vivo validation precludes mechanistic assignment: Our data cannot distinguish whether CCBE1 acts in a tumor‐cell‐intrinsic manner (e.g., by promoting EMT and subsequent chemokine secretion) or through microenvironment‐mediated remodeling (e.g., endothelial–macrophage crosstalk). While our findings add novel candidate mechanisms beyond known CRC macrophage biology, specifically implicating CCBE1 as a potential hub linking tumor‐derived signals to protumoral macrophage infiltration, experimental models (e.g., CCBE1 knockdown/overexpression in syngeneic or xenograft settings) are required to establish causality and cell‐type‐specific function. Additionally, the Cox and LASSO regressions were employed as a variable screening strategy rather than a formal prognostic model; therefore, internal cross‐validation and external cohort validation were not performed. Future studies should develop a dedicated prognostic model incorporating CCBE1, along with functional in vivo experiments to delineate its mode of action. By explicitly acknowledging these factors, particularly purity/confounding, algorithmic uncertainty, and absence of in vivo validation, we aim to provide a realistic foundation for subsequent mechanistic and translational investigations.

## Funding

No funding was received for this research.

## Ethics Statement

The authors have nothing to report.

## Consent

The authors have nothing to report.

## Conflicts of Interest

The authors declare no conflicts of interest.

## Supporting Information

Additional supporting information can be found online in the Supporting Information section.

## Supporting information


**Supporting Information 1** Figure S1. Identification of M2 macrophages by three deconvolution algorithms.

**Supporting Information 2** Figure S2. Correlation between CCBE1 and M2 macrophage after purity adjustment.

**Supporting Information 3** Figure S3. Correlation between CCBE1 expression and tumor purity.

**Supporting Information 4** Figure S4. CCBE1 expression in colorectal cancer cell lines and validation of shRNA‐mediated knockdown. A: qPCR analysis showing upregulation of CCBE1 in colon cancer cells compared to normal NCM460 cells. B: Efficiency of CCBE1 knockdown demonstrated through qPCR assays.

## Data Availability

The datasets used and/or analyzed during the current study are available from the corresponding author on reasonable request (https://doi.org/10.6084/m9.figshare.32229018).
