# Supplementary figures and images for: Comprehensive Analysis of Macrophage Dynamics, CCBE1, and Their Implications in Colorectal Cancer Microenvironment: Insights Into Tumor Progression and Therapeutic Opportunities
Source: Genet Res (Camb). 2026 Jul 1;2026:2678696. doi: 10.1155/genr/2678696 (PMC13319896; doi:10.1155/genr/2678696)

■ CIBERSORT ■ QUANTISEQ ■ XCELL

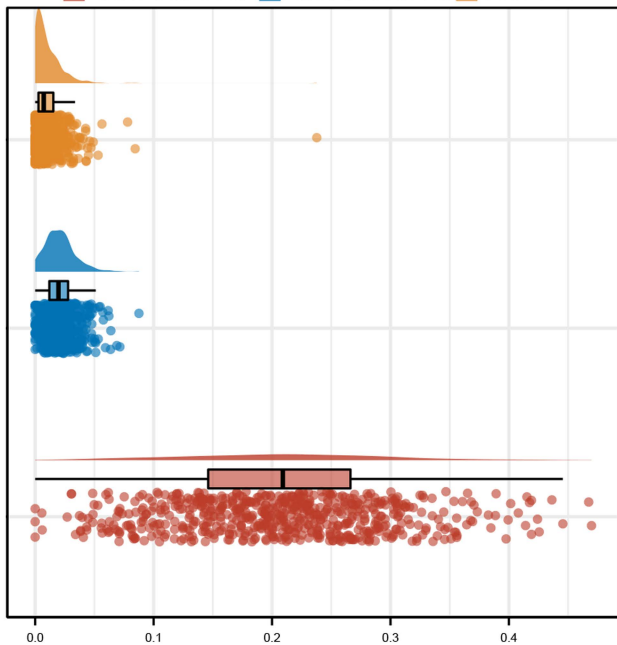

Supplement: Supplementary file 1 — Supporting Information 1 Figure S1. Identification of M2 macrophages by three deconvolution algorithms. [file GENR-2026-2678696-s001.pdf]

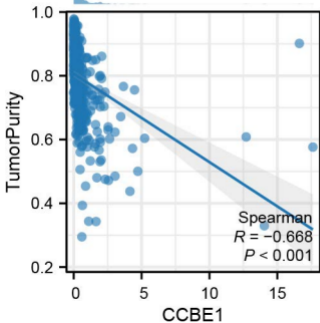

Supplement: Supplementary file 2 — Supporting Information 2 Figure S2. Correlation between CCBE1 and M2 macrophage after purity adjustment. [file GENR-2026-2678696-s002.pdf]

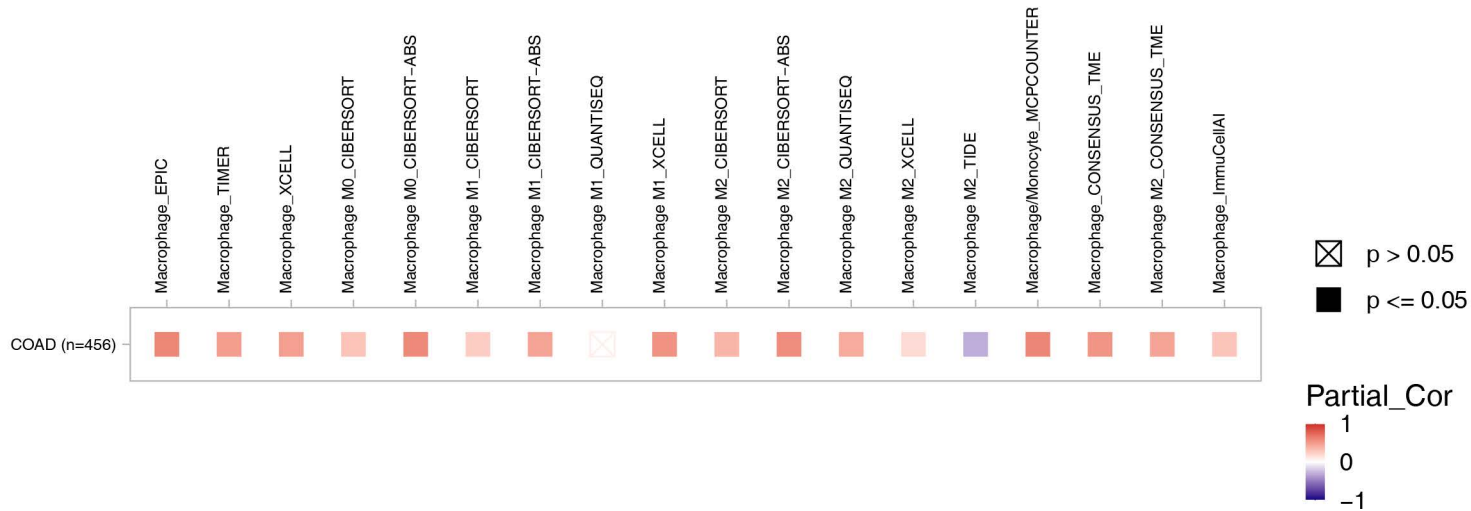

Supplement: Supplementary file 3 — Supporting Information 3 Figure S3. Correlation between CCBE1 expression and tumor purity. [file GENR-2026-2678696-s003.pdf]

A

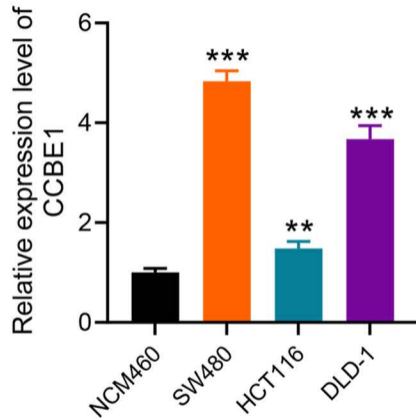

B

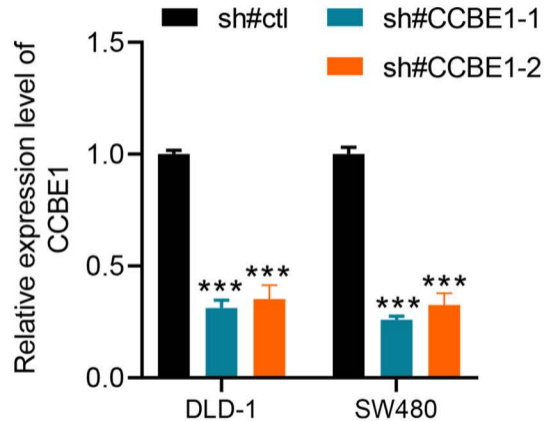

Supplement: Supplementary file 4 — Supporting Information 4 Figure S4. CCBE1 expression in colorectal cancer cell lines and validation of shRNA‐mediated knockdown. A: qPCR analysis showing upregulation of CCBE1 in colon cancer cells compared to normal NCM460 cells. B: Efficiency of CCBE1 knockdown demonstrated through qPCR assays. [file GENR-2026-2678696-s004.pdf]
